# Scale-Dependent Effects of Growth Stage and Elevational Gradient on Rice Phyllosphere Bacterial and Fungal Microbial Patterns in the Terrace Field

**DOI:** 10.3389/fpls.2021.766128

**Published:** 2022-01-14

**Authors:** Pei Wang, Jianping Dai, Luyun Luo, Yong Liu, Decai Jin, Zhuo Zhang, Xiaojuan Li, Wei Fu, Tao Tang, Youlun Xiao, Yang Hu, Erming Liu

**Affiliations:** ^1^State Key Laboratory of Hybrid Rice, Institute of Plant Protection, Hunan Academy of Agricultural Sciences, Changsha, China; ^2^College of Plant Protection, Hunan Agricultural University, Changsha, China; ^3^Southern Regional Collaborative Innovation Center for Grain and Oil Crops in China, Changsha, China; ^4^Yangtze Normal University, Chongqing, China; ^5^Chinese Academy of Sciences Key Laboratory of Environmental Biotechnology, Research Center for Eco-Environmental Sciences, Chinese Academy of Sciences, Beijing, China; ^6^Zhejiang Academy of Forestry, Hangzhou, China

**Keywords:** rice phyllosphere, elevational gradient, growth stage, fungi, bacteria

## Abstract

The variation of phyllosphere bacterial and fungal communities along elevation gradients may provide a potential link with temperature, which corresponds to an elevation over short geographic distances. At the same time, the plant growth stage is also an important factor affecting phyllosphere microorganisms. Understanding microbiological diversity over changes in elevation and among plant growth stages is important for developing crop growth ecological theories. Thus, we investigated variations in the composition of the rice phyllosphere bacterial and fungal communities at five sites along an elevation gradient from 580 to 980 m above sea level (asl) in the Ziquejie Mountain at the seedling, heading, and mature stages, using high-throughput Illumina sequencing methods. The results revealed that the dominant bacterial phyla were Proteobacteria, Actinobacteria, and Bacteroidetes, and the dominant fungal phyla were Ascomycota and Basidiomycota, which varied significantly at different elevation sites and growth stages. Elevation had a greater effect on the α diversity of phyllosphere bacteria than on that phyllosphere fungi. Meanwhile, the growth stage had a great effect on the α diversity of both phyllosphere bacteria and fungi. Our results also showed that the composition of bacterial and fungal communities varied significantly along elevation within the different growth stages, in terms of both changes in the relative abundance of species, and that the variations in bacterial and fungal composition were well correlated with variations in the average elevation. A total of 18 bacterial and 24 fungal genera were significantly correlated with elevational gradient, displaying large differences at the various growth stages. Soluble protein (SP) shared a strong positive correlation with bacterial and fungal communities (*p* < 0.05) and had a strong significant negative correlation with *Serratia*, *Passalora*, unclassified_Trichosphaeriales, and antioxidant enzymes (*R* > 0.5, *p* < 0.05), and significant positive correlation with the fungal genera *Xylaria*, *Gibberella*, and *Penicillium* (*R* > 0.5, *p* < 0.05). Therefore, it suggests that elevation and growth stage might alter both the diversity and abundance of phyllosphere bacterial and fungal populations.

## Introduction

The phyllosphere refers to aboveground plant surfaces as a habitat for plant associated microbes, including bacteria, fungi, protists, and viruses (Vorholt, 2012; Bringel and Couée, 2015). In comparison with coastal seawater habitats and farm soil, leaves are believed to represent one of the largest microbial habitats on Earth but is an environment of reduced bacterial complexity (Delmotte et al., 2009), dominated by a few bacterial phyla, including Actinobacteria, Bacteroidetes, Firmicutes, and Proteobacteria. The phyllosphere is influenced by different environmental factors (Knief et al., 2010; Wellner et al., 2011; Rastogi et al., 2012; Copeland et al., 2015; Ding and Melcher, 2016), where the inhabitants face harsh environmental conditions including ultraviolet (UV) radiation, low free water availability, and nutrient limitations (Colla et al., 2017; Wang and Cernava, 2020). Phyllosphere microbial communities can affect global carbon and nitrogen cycles (Whipps et al., 2008). Individuals within this community may be beneficial, pathogenic, or antagonistic for the host plant and can strongly affect plant health (Vorholt, 2012; Brader et al., 2017; Matsumoto et al., 2021).

Elevational gradients strongly affect microbial biodiversity in the rhizosphere and bulk soil by altering plant and soil properties. The potential response of fungal assemblages to climate change has been investigated principally in soil systems (Sheik et al., 2011; Yuste et al., 2011; Bahram et al., 2015). Guo et al. (2020) suggested that bacterial and fungal α diversity were significantly higher at mid-elevation, while arbuscular mycorrhizal fungi (AMF) α diversity decreased monotonically, and that the beta diversities of the three groups were significantly affected by elevational gradients. Tang et al. (2020) highlighted that elevation was the main predictor to determine rhizosphere microbial community structure in the alpine tundra of the Changbai Mountains. Zhu et al. (2020) showed that seasonal dynamics of bacterial communities were much weaker than those imposed by elevation. However, there have been few studies on the influence of elevation on phyllosphere microorganisms. Cordier et al. (2012a) showed that the composition of fungal assemblages varied significantly over an elevational gradient, suggesting that climate warming might alter both the diversity and abundance of phyllosphere fungal species. Elevation, which is significantly related to mean annual temperature, is also known to affect the community composition of phyllosphere fungi (Qian et al., 2018). The potential responses of phyllosphere bacterial assemblages to elevational gradients have been much less thoroughly explored.

In this study, we investigated the effects of elevation and growth stage on the composition of rice phyllosphere bacterial and fungal communities. We used high-throughput Illumina sequencing to test the following two hypotheses: (1) the diversity and composition of phyllosphere bacterial and fungal communities would change with elevation at the same growth stages and (2) variation in the diversity and composition of phyllosphere bacterial and fungal communities within different growth stages at the same elevation.

## Materials and Methods

### Experimental Design and Site Description

The study was conducted along an elevational gradient extending from 500 to 1,200 m asl at Ziquejie Mountain, with a special terraced structure, in the County Xinhua of the Hunan province, China. We selected five sites (580, 680, 780, 880, and 980 m asl) along with this elevation range that contained a high proportion of rice (Feng Lian 1) grown on the same slope in order to avoid differences in solar exposure. At each elevation, we defined six plots located about 80 m apart. Within each plot, we selected five sub-plots that were located close together. We sampled two leaves per plant located on the outside of the stalk. The rice leaves were sampled three times [July 20 (seedling stage), August 20 (heading stage), and September 12 (mature stage)] in 2018, and each sampling process was completed within 1 day. The leaves were placed in individual plastic bags and transported back to the laboratory at 4°C and stored at that temperature until DNA extraction. Phyllosphere microorganisms were collected as previously described (Delmotte et al., 2009; Redford and Fierer, 2009; Xie et al., 2015), with slight modifications. In brief, 10 g of leaf was submerged in 100 ml of phosphate-buffered saline (PBS) with 0.01% Tween-80 in a 250-ml sterile conical flask. The flask was shaken at 250 rpm for 30 min at 28°C, and then subjected to ultrasonication for 10 min. The microbes were then collected by a 0.22-μm filter using air pump filtration, and the microfiltration membranes were stored at −80°C for subsequent DNA extraction.

### Determination of Total Soluble Protein and Enzyme Activity of Rice Leaves

About 0.1 g of rice leaves was weighed and then transferred to a 1.5-ml centrifuge tube with magnetic beads, 20 mM PBS (pH = 7.4) was added at nine times the weight of the rice leaves, and the leaves were ground into a homogenate with a tissue grinder. The samples were centrifuged at 3,500 rpm for 15 min, and the supernatant of the samples was taken to obtain 10% homogenate for subsequent analysis.

The content of total soluble protein (SP) was measured by Coomassie brilliant blue method. The activity of antioxidant enzymes (CAT: catalase, SOD: superoxide dismutase, and POD: peroxidase) of the rice leaves were measured at different growth stages and elevations in the study. The rice leaves samples were detected using the Biochemical Kit (Nanjing Jiancheng Biotechnology Institute, Nanjing, China) according to the manufacturer’s protocol.

### DNA Extraction and Purification

The rice phyllosphere DNA samples were extracted using the MP FastDNA^®^ SPIN Kit for soil (MP Biomedicals, Solon, OH, United States) according to the manufacturer’s protocol. Before PCR amplification, the DNA concentration was measured and diluted to 30 ng/ml. The V5–V6 region of the bacterial 16S rRNA gene was amplified using the primer set 799F (AACMGGATTAGATACCCKG) and 1115R (AGGGTTGCGCTCGTTG), while the internally transcribed spacer (ITS) region of fungi was amplified with primer pair gITS7F (GTGARTCATCGARTCTTTG)/ITS4 (TCCTCCGCTTATTGATATGC), each primer pair contained a unique 12 nt barcode, and was amplified as previously described by Kong et al. (2018). The PCR products were separated by agarose gel electrophoresis, and the strong bands at ∼300 bp were collected and purified with an E.Z.N.A.^®^ Gel Extraction Kit (Omega Bio-tek, Norcross, GA, United States). The PCR products were quantified using Qubit™ 2 Fluorometer and paired-end sequenced (2 × 250 bp) on an Illumina MiSeq platform by Annoroad Gene Technology Co., Ltd. (Beijing, China) according to the standard protocols.

### 16S rRNA Gene Amplification, Sequencing, and Processing

The 16S rDNA and ITS raw sequence data reads, in fastq format, were collected and processed with an in-house pipeline^1^ containing a series of bioinformatics tools. First, samples were separated based on the 12-bp barcodes and primers. Paired end reads were combined using the FLASH program developed by Magoc and Salzberg (2011). Sequences containing ambiguous (N) bases were removed. Chimera sequences also were detected and removed using UPARSE developed by Edgar (2013)^2^. All sequences were clustered and operational taxonomic units (OTUs) were generated at the 97% similarity level, low abundance OTUs (≤1 counts) were removed from the OTU table. All chloroplast and mitochondrial sequences were removed. Representative sequences for each OTU were assigned to taxonomic groups using the RDP Classifier database (RDP training set RDP to release 11.5 database and unite database 8.2 version) (Wang et al., 2007). After accounting for sampling depth, we obtained a resampled OTU table with 12,477 and 977 sequences for bacterial and fungal samples, respectively, for the subsequent analyses. All raw bacterial sequences were deposited in the SRA database short-read archive under accession number PRJNA675674. Principal coordinate analysis (PCoA) based on the Bray–Curtis distance was performed in subsequent analyses using the “vegan” package in R (v.3.2.5). Differences in microbial community composition between two groups were evaluated by using non-parametric multi-response permutation procedure (MRPP), analysis of similarities (ANOSIM), and non-parametric permutational multivariate analysis of variance of the Adonis function (ADONIS) using the “vegan” package in R (v.3.2.5) (Anderson, 2001; Dixon, 2010). Mantel test, canonical correlation analysis (CCA), and CCA-based variance partitioning analysis (VPA) were used to measure the variations in different environmental factors.

### Statistical Analysis

The statistical significance of differences between α diversity indices of different groups and the relative abundance of the taxonomic subgroups were assessed by performing a one-way ANOVA followed by Duncan’s multiple range test. Correlation analyses between elevation and the top 30 genera were also conducted with the Spearman method using the IBM SPSS software for Windows, version 22.0 (IBM Corp., Armonk, NY, United States).

## Results

### The Physical and Chemical Properties of Rice Leaves

**FIGURE 1:**
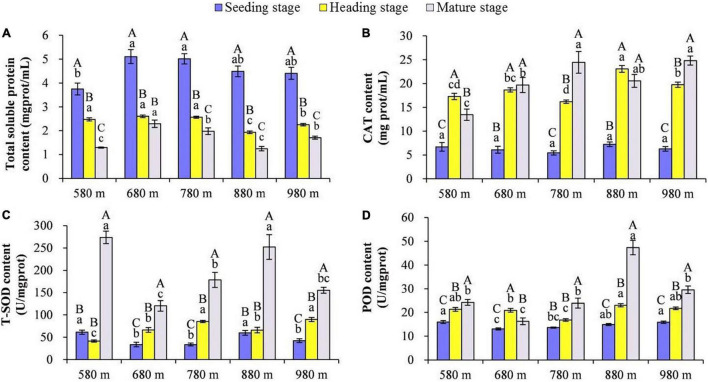
The effect of elevation and growth stage on total soluble protein and activity of antioxidant enzymes. **(A)** Total soluble protein. **(B)** CAT content. **(C)** T-SOD content. **(D)** POD content. Capital letters indicate significant differences between different growth stages at the same elevation. Lowercase letters indicate significant differences between different elevations at the same growth stage. SP, soluble protein; CAT, catalase; SOD, superoxide dismutase; POD, peroxidase.

The content of total SP, CAT, SOD, and POD in rice leaves at different elevations and growth stages was compared (Figures 1A–D). The results showed that at the same elevation, except for 680 m asl, the total SP in rice leaves displayed significant differences among the different growth stages, with the seedling stage > heading stage > mature stage. There was no significant difference from 580 to 780 m asl for the total SP content at the heading stage, but it was significantly higher than at 880 and 980 m asl. The content of total SP was the highest at 680 m asl, followed by 780 and 980 m asl, and the lowest at 580 and 880 m asl.

At 580 m asl, the CAT content of rice leaves was heading stage > mature stage > seedling stage. At 680 and 880 m asl, there was no significant difference at the heading stage and mature stage, respectively, but the content of both stages was significantly higher than the seedling stage. At 780 and 980 m asl, the CAT content of rice leaves was a mature stage > heading stage > seedling stage. The CAT content in rice leaves at different elevations during the same growth stage was also compared, and no significant difference was found for the five elevations at the seedling stage. At the heading stage, the CAT content in rice leaves at higher elevations (880 and 980 m asl) was significantly higher than that at 580–780 m asl. Finally, at the mature stage, for elevations from 780 to 980 m asl, the CAT content was significantly higher than at lower elevations (from 580 to 680 m asl). The SOD content of rice leaves at the mature stage was significantly higher than at the seedling and heading stages at the elevations of 580 and 880 m asl. At the mature stage, for the elevations of 680, 780, and 980 m asl, the SOD content of rice leaves was a mature stage > heading stage > seedling stage. The SOD content of rice leaves at 580 and 880 m asl was significantly higher than at the other three elevations (680, 780, and 980 m asl) at the seedling and mature stages. The SOD content of rice leaves was significantly higher at the elevations of 780 and 980 m asl than the other three elevations (580, 680, and 880 m asl) at the heading stage. At the elevations of 580, 880, and 980 m asl, the POD content of rice leaves was a mature stage > heading stage > seedling stage, and at the elevation of 680 m asl, the POD content of rice leaves was heading stage > mature stage > seedling stage. While at the elevation of 780 m asl, the POD content of rice leaves in the mature stage was significantly higher than the seedling stage and heading stage. These results showed that the POD content in rice leaves at elevations of 580, 880, and 980 m asl was significantly higher than elevations of 680 and 780 m asl at the seedling and heading stages. At the mature stage, the POD content in rice leaves had the highest value at 880 m asl, followed by 580, 780, and 980 m asl, with the lowest at 680 m asl.

### The Variation of the Rice Phyllosphere Bacterial and Fungal α Diversity

**FIGURE 2:**
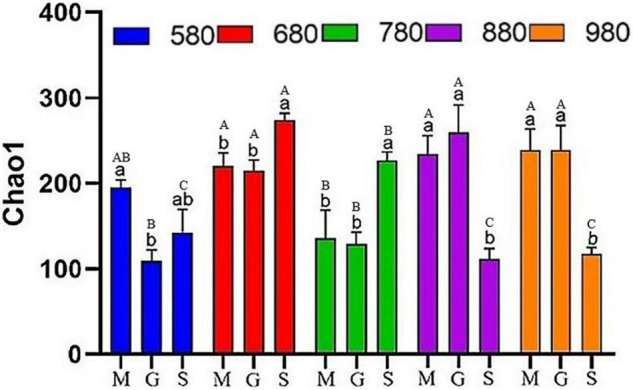
The α diversity indices of phyllosphere bacteria among different elevations and growth stages. Comparison of α diversity of rice phyllosphere bacteria at different growth stages at the same elevation, M: seedling stage, G: heading stage, S: mature stage. Capital letters indicate significant differences between different growth stages at the same growth stage. Lowercase letters indicate significant differences at different elevations at the same elevation.

In this study, rice phyllosphere bacterial and fungal α diversity (Chao1) at different growth stages along an elevational gradient was analyzed. The results of α diversity analysis for the rice phyllosphere bacterial community are shown in Figure 2. At 580 m asl, Chao1 first decreased and then increased with rice growth progression. There was no significant difference between the seedling stage and heading stage, but the mature stage increased significantly at 680 and 780 m asl, while decreasing significantly at 880 and 980 m asl. These results indicated that the variation trend of bacterial Chao1 differed with the growth stage at different elevations. The α diversity at different elevations during the seeding, heading, and maturity stages was compared and analyzed (Figure 2). At the seedling and heading stage, Chao1 first increased from 580 to 680 m asl, then decreased from 680 to 780 m asl, and then increased again gradually from 780 to 980 m asl. At the mature stage, Chao1 increased first and then decreased gradually, reaching its peak value at 680 m asl. The results showed that, from 680 to 980 m asl, there was no significant difference in rice seedling and heading stages, while diversity in the mature stage was different. In addition, the variation trend of diversity at the seedling stage and heading stage was similar at different elevations. Overall, the diversity increased gradually with elevation, while the diversity decreased gradually from 680 to 980 m asl at the mature stage.

**FIGURE 3:**
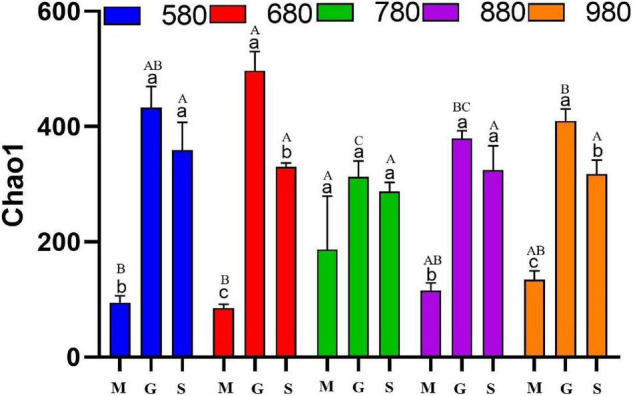
The α diversity indices of phyllosphere fungi among different elevations and growth stages. Comparison of α diversity of rice phyllosphere fungi at different growth stages at the same elevation, M: seedling stage, G: heading stage, S: mature stage. Capital letters indicate significant differences between different growth stages at the same growth stage. Lowercase letters indicate significant differences at different elevations at the same elevation.

The analysis of rice phyllosphere fungal community α diversity is shown in Figure 3. The results indicated that fungal Chao1 at 580 and 880 m asl at the seedling stage was significantly lower than that at the heading and maturity stages, and there was no significant difference between the heading stage and maturity stage. At 680 and 980 m asl, fungal Chao1 first increased and then decreased, with significant differences among the three stages. There was no significant difference between the three stages at 780 m asl. The results showed that the phyllosphere fungal α diversity at the seedling stage increased gradually with the increase of elevation between 580 and 980 m asl. At the heading stage, the fungal α diversity first increased (580–680 m asl) and appears to have peaked at 780 m asl and then decreased. These results showed that rice phyllosphere fungal Chao1 at different elevations and stages had the same variation trend, first increasing and then decreasing. At the heading stage, the fungal α diversity of Chao1 was higher than other two stages. The fungal α diversity of rice phyllosphere in the same growth stage had no significant change with elevations.

### Operational Taxonomic Unit Analysis

The shared and unique OTU numbers are shown in the Venn diagrams of Supplementary Figure 1. With a 97% similarity cutoff, a total of 998 and 977 OTUs were obtained for bacterial and fungal sequences, respectively. At the seedling stage, there were 88 shared bacterial OTUs among the five elevation groups. The 780 m asl (48, 22.02%) group had the highest number of unique OTUs, while the 580 m asl (25, 8.77%) group had the lowest number of unique bacterial OTUs. At the heading stage, 79 bacterial OTUs were shared among the five elevation groups. The 880 m asl (136, 30.49%) group had the highest number of unique OTUs, and the 580 m asl (8, 4.88%) group had the lowest number of unique OTUs, respectively. At the mature stage, there were 80 shared bacterial OTUs among the five elevation groups. The 680 m asl (95, 26.61%) group had the highest number of unique OTUs, and the 880 m asl (9, 5.45%) group had the lowest number of unique OTUs, respectively.

For the rice phyllosphere fungal community at the seedling stage, there were a total of 563 OTUs across the five elevation sample groups, with the greatest number of unique OTUs in the 780 m group (221, 39.25%), and the lowest number of unique OTUs in the 680 m group (10, 1.78%), with a total of 61 shared OTUs (Supplementary Figure 1D). At the heading stage, the phyllosphere fungal community was composed of 854 OTUs across the five elevation groups. The number of unique OTUs was highest in the 680 m group (70, 8.20%), and lowest in the 780 m group (13, 1.52%), with a total of 222 OTUs shared between all groups (Supplementary Figure 1E). At the maturity stage, there were a total of 806 OTUs in the phyllosphere fungal community across the five elevations, with the 580 m group possessing the highest number of unique OTUs (67, 8.31%), while 780 m group had the lowest number of unique OTUs (19, 2.36%), with a total of 198 OTUs shared between all groups (Supplementary Figure 1F).

### Composition of the Rice Phyllosphere Bacterial and Fungal Community

**FIGURE 4:**
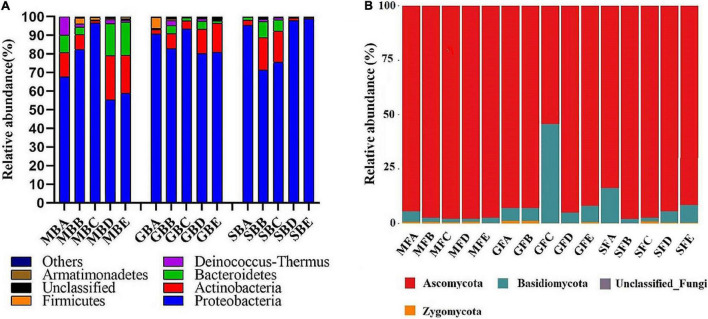
The relative abundance of the dominant phyllosphere bacterial **(A)** and fungal **(B)** population at the phylum level. B, bacteria; F, fungi. M, seedling stage; G, heading stage; S, mature stage. (A) 580 m asl; (B) 680 m asl; (C) 780 m asl; (D) 880 m asl; (E) 980 m asl.

At the phylum level, a total of 19 bacterial phyla and 1 archaeal phylum were identified. The dominant bacterial populations (relative abundance greater than 4%) were Proteobacteria, Actinobacteria, and Bacteroidetes in the seeding, heading, and mature stages of rice phyllosphere samples (Figure 4A). At the seedling stage, the relative abundance of Proteobacteria first increased and then decreased with elevation, while that of Actinobacteria and Bacteroidetes first decreased and then increased, with the turning point being 780 m asl. At the heading stage, the highest relative abundance of Proteobacteria was at 780 m asl, while that of Actinobacteria at 880 m and 980 m asl was higher than other elevations. At the mature stage, the relative abundance of Proteobacteria first decreased and then increased with elevation, while that of Bacteroidetes first increased and then decreased.

A total of four phyla were identified among the phyllosphere fungi (Figure 4B). Ascomycota and Basidiomycota were the dominant fungi in rice phyllosphere samples at the seedling, heading, and maturity stages. There were significant differences at the phylum level at different elevations during the same growth stage. With the increasing elevation at the seedling stage, the relative abundance of Ascomycota increased, while that of Basidiomycota decreased. At the heading stage, the relative abundance of Ascomycota first decreased and then increased, while Basidiomycota displayed the opposite trend with an inflection point at 780 m asl. At the mature stage, the relative abundance of Basidiomycota first decreased and then increased with the increase of elevation.

At the genus level, we analyzed the correlation between the top 30 bacterial and fungal genera and elevation for each of the three growth stages (Table 1). At the seedling stage, the elevation was positively correlated with the bacterial genera *Curtobacterium* and *Mucilaginibacter*, and negatively correlated with *Acinetobacter* and *Pseudomonas* (*R* > 0, *p* < 0.05), while lower correlations were observed with fungal genera. At the heading stage, the elevation was significantly positively correlated with the bacterial genera *Lapillicoccus*, *Methylobacterium*, *Mucilaginibacter*, *Acidovorax*, *Rhizobium*, *Microbacterium*, *Pedobacter*, and *Aureimonas*, and negatively correlated with *Pantoea*, *Pseudomonas*, and *Buttiauxella* (*R* > 0, *p* < 0.05), while the elevation was significantly positively correlated with the fungal genera *Alternaria*, Phaeosphaeriaceae_unclassified, and unclassified_Trichosphaeriales, and negatively correlated with *Passalora*, Mycosphaerellaceae_unclassified, and *Periconia* (*R* > 0, *p* < 0.05). While at the mature stage, the elevation was negatively correlated with the bacterial genera *Moraxella*, *Lapillicoccus*, *Spirosoma*, *Methylobacterium*, *Kineococcus*, *Geodermatophilus*, and *Nakamurella* (*R* > 0, *p* < 0.05), and positively correlated with the fungal genera *Passalora*, *Periconia*, and Mycosphaerellaceae_unclassified, and negatively correlated with the fungal genera Phaeosphaeriaceae_unclassified, Dothideomycetes_unclassified, and *Alternaria*.

**TABLE 1:** The Spearman correlation analysis between top 30 rice phyllosphere bacterial and fungal populations and elevation.

	Genus	Seeding stage	Heading stage	Mature stage
Bacteria	*Acidovorax*	−0.0109	0.738***	−0.233
	*Acinetobacter*	−0.565**	−0.174	−0.342
	*Aureimonas*	0.0695	0.601***	0.00545
	*Buttiauxella*	−0.0844	−0.746***	0.0381
	*Curtobacterium*	0.582***	−0.11	−0.38*
	*Geodermatophilus*	0.354	0.176	−0.525**
	*Kineococcus*	0.185	0.317	−0.531**
	*Lapillicoccus*	0.437*	0.511**	−0.719***
	*Methylobacterium*	0.392*	0.576***	−0.553**
	*Microbacterium*	−0.0735	0.733***	−0.169
	*Moraxella*	−0.246	−0.168	−0.722***
	*Mucilaginibacter*	0.502**	0.666***	−0.271
	*Nakamurella*	0.485**	0.254	−0.514**
	*Pantoea*	0.485**	−0.692***	−0.484**
	*Pedobacter*	−0.142	0.615***	−0.0558
	*Pseudomonas*	−0.584***	−0.515**	−0.0926
	*Rhizobium*	−0.46*	0.674***	0.225
	*Spirosoma*	0.473**	0.106	−0.622***
Fungi	Phaeosphaeriaceae_unclassified	−0.187	0.792**	−0.902**
	*Passalora*	−0.0963	−0.945**	0.847**
	Dothideomycetes_unclassified	−0.166	−0.18	−0.812**
	*Alternaria*	0.182	0.604**	−0.792**
	Mycosphaerellaceae_unclassified	0.421**	−0.824**	0.743**
	Pleosporales_unclassified	−0.154	0.267	−0.69**
	Unclassified_Trichosphaeriales	−0.412**	0.812**	−0.682**
	*Periconia*	0.362*	−0.698**	0.635**
	*Ophiosphaerella*	−0.296	0.188	−0.612**
	Nectriaceae_unclassified	−0.207	−0.349	−0.584**
	Unclassified_Montagnulaceae	0.217	−0.859**	0.549**
	*Acremonium*	−0.347*	0.0941	−0.518**
	*Phoma*	0.196	−0.361	0.475*
	Ascomycota_unclassified	0.0385	−0.588**	0.459*
	Unclassified_Pleosporales	0.121	0.0471	−0.424*
	*Monographella*	0.449**	−0.188	0.345
	*Nigrospora*	−0.17	−0.514**	0.337
	*Fusarium*	0.196	−0.502*	−0.286
	*Cryptococcus*	−0.327*	−0.749**	0.188
	*Paraphaeosphaeria*	−0.326*	−0.404*	−0.106
	*Aspergillus*	−0.24	−0.498*	−0.0981
	*Pyrenochaetopsis*	0.291	0.494*	0.0942
	*Penicillium*	0.00342	−0.651**	0.00784
	*Cladosporium*	−0.456**	−0.0941	−0.00392

*p < 0.05, **p < 0.01, ***p < 0.001.

We analyzed the dynamic changes of bacterial genera that displayed a significant correlation with elevation at the different sites during the seedling, heading, and mature stages (Table 2). At the seedling stage, the relative abundances of *Acinetobacter* and *Pseudomonas* at 580 m asl were significantly higher than other elevations, but there was no significant difference among the other four elevations. The relative abundances of *Curtobacterium* and *Mucilaginibacter* at 580, 680, and 780 m asl were significantly lower than at 880 and 980 m asl. At the heading stage, the relative abundances of *Acidovorax* and *Microbacterium* were significantly higher at 980 m asl than at the other four elevations. While the relative abundance of *Buttiauxella* at 580, 680, and 780 m asl was significantly higher than at 880 and 980 m asl, and the relative abundance of *Mucilaginibacter* at 580, 680, and 780 m asl was significantly lower than at 880 and 980 m asl. The relative abundance of *Pantoea* at 580 and 680 m asl was higher than that between 780 and 980 m, while *Rhizobium* and *Lapillicoccus* displayed no significant difference among the five elevations. At the mature stage, the relative abundances of *Moraxella*, *Lapillicoccus*, *Spirosoma*, *Methylobacterium*, *Kineococcus*, *Geodermatophilus*, and *Nakamurella* first increased and then decreased with elevation, reaching a maximum value at 680 m asl, which was consistent with the trend of α diversity.

**TABLE 2:** The relative abundance of genera was significantly correlated with elevation among the top 30 phyllosphere bacterial genera.

Growth stage	Genus	580 m	680 m	780 m	880 m	980 m
Seeding stage	*Acinetobacter*	4.15 ± 1.81a	0.47 ± 0.19b	0.12 ± 0.03b	1.76 ± 1.65ab	0.08 ± 0.02b
	*Curtobacterium*	1.51 ± 0.34b	1.68 ± 0.72b	0.48 ± 0.25b	4.82 ± 0.43a	5.14 ± 0.63a
	*Mucilaginibacter*	0.21 ± 0.06b	0.27 ± 0.18b	0.10 ± 0.10b	1.55 ± 0.34a	1.72 ± 0.42a
	*Pseudomonas*	27.34 ± 8.89a	8.96 ± 2.6b	0.19 ± 0.03b	1.26 ± 0.36b	1.14 ± 0.81b
Heading stage	*Acidovorax*	0.02 ± 0.01b	0.07 ± 0.02b	0.06 ± 0.01b	0.12 ± 0.05b	1.56 ± 0.32a
	*Aureimonas*	0.02 ± 0.01c	0.61 ± 0.02b	0.36 ± 0.02bc	0.76 ± 0.22ab	1.23 ± 0.36a
	*Buttiauxella*	70.01 ± 3.45a	51.7 ± 6.8b	63.16 ± 6.1ab	18.02 ± 4.97c	21.28 ± 1.98c
	*Methylobacterium*	0.43 ± 0.12b	3.59 ± 0.87ab	1.12 ± 0.09b	7.04 ± 2.61ab	3.22 ± 0.48a
	*Microbacterium*	0.30 ± 0.08b	2.52 ± 0.38b	1.95 ± 0.17b	2.19 ± 0.78b	11.17 ± 1.69a
	*Mucilaginibacter*	0.06 ± 0.03b	0.04 ± 0.01b	0.06 ± 0.03b	0.30 ± 0.10a	0.26 ± 0.03a
	*Pantoea*	4.57 ± 2.46a	2.47 ± 0.93ab	0.77 ± 0.17b	0.23 ± 0.09b	0.14 ± 0.02b
	*Pedobacter*	0.01 ± 0.01b	0.02 ± 0.01b	0.25 ± 0.08a	0.11 ± 0.08ab	0.13 ± 0.03ab
	*Pseudomonas*	1.27 ± 0.52c	6.43 ± 0.52a	2.8 ± 0.35b	0.46 ± 0.24c	0.36 ± 0.04c
	*Rhizobium*	0.00 ± 0.00a	0.00 ± 0.00a	0.00 ± 0.00a	0.02 ± 0.01a	1.90 ± 1.63a
	*Lapillicoccus*	0.01 ± 0.00a	0.02 ± 0.00a	0.00 ± 0.00a	0.44 ± 0.30a	0.12 ± 0.04a
Mature stage	*Geodermatophilus*	0.10 ± 0.03b	0.76 ± 0.14a	0.57 ± 0.16a	0.05 ± 0.01b	0.02 ± 0.01b
	*Kineococcus*	0.50 ± 0.17c	3.57 ± 0.22a	2.05 ± 0.25b	0.09 ± 0.03c	0.09 ± 0.01c
	*Methylobacterium*	1.20 ± 0.39c	8.48 ± 0.77a	3.76 ± 0.42b	0.77 ± 0.33c	0.28 ± 0.06c
	*Moraxella*	0.10 ± 0.04b	5.57 ± 1.11a	0.00 ± 0.00a	0.00 ± 0.00a	0.00 ± 0.00a
	*Nakamurella*	0.14 ± 0.07b	0.79 ± 0.45a	0.2 ± 0.07ab	0.05 ± 0.01b	0.01 ± 0.01b
	*Spirosoma*	1.49 ± 0.55b	7.37 ± 2.12a	1.96 ± 0.82b	0.21 ± 0.04b	0.11 ± 0.04b
	*Lapillicoccus*	0.28 ± 0.12b	1.14 ± 0.55a	0.06 ± 0.04b	0.02 ± 0.00b	0.01 ± 0.00b

*Different letter indicated significant difference among different groups.*

We also analyzed the dynamic changes of fungal genera that displayed a significant correlation with elevation at the different sites during the seedling, heading, and mature stages (Table 3). At the seedling stage, the relative abundance of the top 30 fungal genera had a low correlation with elevation. At the heading stage, the relative abundance of most fungal genera was not significantly different at the five elevations, the exception being unclassified_Trichosphaeriales, which was significantly higher at 680 m than at 980 m asl, and no significant difference was found between the other elevations. At the mature stage, the relative abundance of most genera showed no significant difference between the five elevations, while the relative abundance of *Acremonium* at 580 m asl was significantly higher than that at 880 m, but there was no significant difference at the other elevations. The relative abundance of *Ophiosphaerella* at 680 m asl was significantly higher than that at 780, 880, and 980 m asl, while no significant difference was found with the other elevations. The relative abundance of unclassified_Trichosphaeriales was significantly higher at 680 m asl than that at 980 m asl, and no significant difference was found at the other elevations.

**TABLE 3:** The relative abundance of genera was significantly correlated with elevation among the top 30 phyllosphere fungal genera.

Growth stage	Genus	580 m	680 m	780 m	880 m	980 m
Seeding stage	Mycosphaerellaceae_unclassified	0.01 ± 0c	0.02 ± 0c	0.02 ± 0.01c	0.08 ± 0.02b	0.19 ± 0.01a
	*Acremonium*	0.18 ± 0.03a	0.07 ± 0.01a	0.04 ± 0.01a	0.34 ± 0.24a	0.04 ± 0.03a
	*Cladosporium*	1.95 ± 0.37c	5.98 ± 0.24a	1.28 ± 0.05c	3.94 ± 0.82b	1.95 ± 0.18c
	*Cryptococcus*	0.39 ± 0.2b	0.32 ± 0.08b	0.03 ± 0.02b	1.66 ± 0.65a	0.49 ± 0.21b
	*Monographella*	0.06 ± 0.01a	0.03 ± 0.01a	0.06 ± 0.02a	0.15 ± 0.08a	0.09 ± 0.04a
	*Paraphaeosphaeria*	0.34 ± 0.11b	1.37 ± 0.11a	0.58 ± 0.11b	1.04 ± 0.19a	0.3 ± 0.04b
	*Periconia*	0.2 ± 0.03b	0.2 ± 0.03b	0.5 ± 0.14a	0.45 ± 0.08ab	0.53 ± 0.11a
	Unclassified_Trichosphaeriales	16.13 ± 1.11b	32.69 ± 1.18a	17.11 ± 1.51b	11.4 ± 2.14c	5.77 ± 1.6d
Heading stage	Mycosphaerellaceae_unclassified	0.06 ± 0.01a	0.11 ± 0.03a	0.01 ± 0a	0.01 ± 0a	0 ± 0a
	Phaeosphaeriaceae_unclassified	0.14 ± 0.07a	0.21 ± 0.05a	0.33 ± 0.02a	0.77 ± 0.27a	1.45 ± 0.18a
	*Alternaria*	0.47 ± 0.13a	0.39 ± 0.04a	0.34 ± 0.02a	0.82 ± 0.19a	1.78 ± 0.24a
	*Aspergillus*	0.34 ± 0.11a	1.03 ± 0.6a	0 ± 0a	0 ± 0a	0.02 ± 0.01a
	*Cryptococcus*	3.59 ± 2.74a	2.33 ± 1.29a	0.16 ± 0a	0.08 ± 0.02a	0.01 ± 0a
	*Fusarium*	0.49 ± 0.16a	0.76 ± 0.36a	0.03 ± 0a	0.06 ± 0.02a	0.11 ± 0.06a
	*Nigrospora*	0.14 ± 0.02a	0.38 ± 0.1a	0.14 ± 0.01a	0.09 ± 0.02a	0.12 ± 0.02a
	*Paraphaeosphaeria*	0.45 ± 0.2ab	0.96 ± 0.24ab	0.33 ± 0.02a	0.24 ± 0.04ab	0.19 ± 0.04b
	*Passalora*	58.83 ± 4.98a	34.86 ± 4.01a	19.97 ± 0.37a	16.96 ± 1.35a	13.78 ± 1.23a
	*Penicillium*	0.33 ± 0.17a	0.43 ± 0.12a	0.02 ± 0a	0.02 ± 0.01a	0.02 ± 0.01a
	*Periconia*	0.53 ± 0.08a	0.4 ± 0.09a	0.06 ± 0.01a	0.03 ± 0.01a	0.08 ± 0.02a
	*Pyrenochaetopsis*	0.02 ± 0.01ab	0.04 ± 0.01b	0.01 ± 0ab	0.04 ± 0.01a	0.26 ± 0.1ab
	Unclassified_Montagnulaceae	0.08 ± 0.02a	0.04 ± 0.01a	0.01 ± 0a	0.01 ± 0a	0.01 ± 0.01a
	Unclassified_Trichosphaeriales	11.79 ± 2.41ab	29.02 ± 4.43a	25.01 ± 0.38ab	63.17 ± 3.58ab	45.67 ± 2.96b
	Ascomycota_unclassified	0.09 ± 0.04a	0.09 ± 0.02a	0 ± 0a	0.01 ± 0a	0.01 ± 0a
Mature stage	Dothideomycetes_unclassified	1.03 ± 0.29a	1.16 ± 0.32a	0.61 ± 0.42a	0.92 ± 0.33a	0.53 ± 0.16a
	Mycosphaerellaceae_unclassified	0.05 ± 0.05a	0 ± 0a	0.08 ± 0.08a	0.27 ± 0.22a	0.7 ± 0.4a
	Nectriaceae_unclassified	6.75 ± 0.84a	4.63 ± 0.81a	8.15 ± 1.87a	2.3 ± 0.57a	7.98 ± 2.52a
	Phaeosphaeriaceae_unclassified	0.18 ± 0.09a	0.23 ± 0.1a	0.02 ± 0.02a	0.06 ± 0.04a	0.01 ± 0.01a
	*Acremonium*	1.27 ± 0.3a	0.66 ± 0.29ab	0.96 ± 0.51ab	0.33 ± 0.14b	0.42 ± 0.22ab
	*Alternaria*	0.39 ± 0.1a	0.86 ± 0.23a	0.73 ± 0.24a	0.52 ± 0.22a	1.15 ± 0.42a
	*Ophiosphaerella*	0.18 ± 0.08ab	0.33 ± 0.15a	0.01 ± 0.01b	0 ± 0b	0.03 ± 0.02b
	*Passalora*	8.3 ± 1.35a	15.96 ± 4.7a	8.17 ± 3.25a	11.84 ± 2.55a	8.37 ± 3.34a
	*Periconia*	0.14 ± 0.07a	0.23 ± 0.15a	0.84 ± 0.3a	0.61 ± 0.34a	0.74 ± 0.3a
	*Phoma*	0.87 ± 0.19a	2.23 ± 0.78a	1.05 ± 0.51a	3.33 ± 0.88a	2.92 ± 1.5a
	Unclassified_Montagnulaceae	0.08 ± 0.08a	0.12 ± 0.12a	0.07 ± 0.05a	0 ± 0a	0.15 ± 0.09a
	Unclassified_Pleosporales	0.11 ± 0.08a	0.21 ± 0.21a	0.09 ± 0.06a	0.12 ± 0.08a	0.06 ± 0.03a
	Unclassified_Trichosphaeriales	1.59 ± 0.38ab	2.39 ± 0.56a	1.14 ± 0.54ab	1.6 ± 0.49ab	0.27 ± 0.1b
	Pleosporales_unclassified	3.46 ± 0.92a	3.22 ± 1.04a	3.13 ± 1.77a	2.95 ± 0.79a	2.14 ± 0.59a
	Ascomycota_unclassified	0.15 ± 0.09a	0.33 ± 0.2a	1.94 ± 1.89a	0.24 ± 0.16a	0.33 ± 0.28a

*The different letters indicated a significant difference among different groups.*

### Bacterial and Fungal Community Structure in Rice Phyllosphere

**FIGURE 5:**
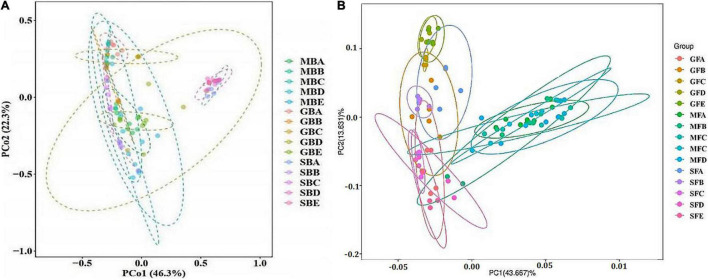
Principal coordinate analysis (PCoA) of rice phyllosphere bacteria **(A)** and fungi **(B)** among different elevational sites and growth stages. **(A)** PCoA of rice phyllosphere bacteria. **(B)** PCoA of rice phyllosphere fungi. B, bacteria; F, fungi. M, seedling stage; G, heading stage; S, mature stage. (A) 580 m asl; (B) 680 m asl; (C) 780 m asl; (D) 880 m asl; (E) 980 m asl.

The results of bacterial PCoA showed that bacteria PCoA1 and PCoA2 accounted for 46.3 and 22.3% of the variation, respectively (Figure 5A). The phyllosphere bacterial communities of rice at the seedling stage and heading stage were relatively similar, while the bacterial community at the mature stage was significantly different from either of the two earlier stages. Fungal PCoA1 and PCoA2 explained 43.667 and 13.631% variation, respectively (Figure 5B). Different from bacteria, there were significant differences among the phyllosphere fungal community at all three stages of rice growth.

To further verify the above results, the differences between groups were compared in the rice bacterial and fungal community structure at different elevations for the three growth stages (Tables 4, 5). The results showed that there was no significant difference in the rice bacterial community between 880 and 980 m asl at the seedling stage, but there were significant differences between the samples of the other elevations. There were significant differences among the five elevations at the heading stage. At the mature stage, 880 m asl was not observed to be significantly different from 580 to 980 m asl, but there were significant differences between other elevations. We also analyzed the community changes in the three growth stages at different elevations, and the results showed that there were significant differences between different growth stages at the same elevation, except for the seedling and heading stages at 680 m asl.

**TABLE 4:** Dissimilarity test of phyllosphere bacterial community between two different sample groups among different elevations at the seedling, heading, and mature stages.

Growth stage		MRPP		ANOSIM		PERMANOVA	
		Delta	*p*	*R*	*p*	*F*	*p*
	580 vs. 680 m	0.4783	0.003	0.4962	0.003	4.0604	0.003
	580 vs. 780 m	0.3007	0.002	0.9574	0.004	23.4574	0.005
	580 vs. 880 m	0.4709	0.01	0.6796	0.005	6.2416	0.007
	580 vs. 980 m	0.4769	0.002	0.7444	0.002	6.4354	0.005
Seeding	680 vs. 780 m	0.333	0.002	0.5129	0.003	6.1696	0.002
stage	680 vs. 880 m	0.5031	0.009	0.5092	0.011	4.69	0.016
	680 vs. 980 m	0.5092	0.007	0.5342	0.012	4.1205	0.004
	780 vs. 880 m	0.3255	0.004	0.8277	0.004	19.1718	0.003
	780 vs. 980 m	0.3316	0.003	0.7518	0.001	15.9034	0.002
	880 vs. 980 m	0.5017	0.058	0.2000	0.067	2.0966	0.079
	580 vs. 680 m	0.2341	0.004	0.5564	0.003	10.9207	0.004
	580 vs. 780 m	0.2511	0.044	0.3074	0.056	3.2379	0.048
	580 vs. 880 m	0.4157	0.002	0.7314	0.003	9.8282	0.003
	580 vs. 980 m	0.2342	0.007	1.0000	0.002	39.8444	0.005
Heading	680 vs. 780 m	0.243	0.005	0.7000	0.003	6.8864	0.002
stage	680 vs. 880 m	0.4075	0.005	0.5685	0.005	6.2698	0.004
	680 vs. 980 m	0.2261	0.004	1.0000	0.003	29.5623	0.003
	780 vs. 880 m	0.4245	0.004	0.5981	0.002	6.5610	0.005
	780 vs. 980 m	0.2431	0.002	1.0000	0.003	26.1789	0.005
	880 vs. 980 m	0.4076	0.005	0.4462	0.002	4.4366	0.003
	580 vs. 680 m	0.1401	0.004	1.0000	0.002	116.8755	0.001
	580 vs. 780 m	0.1739	0.002	1.0000	0.001	119.5832	0.002
	580 vs. 880 m	0.0965	0.264	0.0935	0.163	1.3831	0.247
	580 vs. 980 m	0.075	0.015	0.3722	0.016	3.6047	0.019
Mature	680 vs. 780 m	0.2264	0.003	0.8407	0.008	16.1691	0.001
stage	680 vs. 880 m	0.1491	0.002	1.0000	0.003	112.7412	0.002
	680 vs. 980 m	0.1276	0.004	1.0000	0.003	122.2524	0.002
	780 vs. 880 m	0.1828	0.002	1.0000	0.002	112.2833	0.002
	780 vs. 980 m	0.1614	0.004	1.0000	0.003	121.7608	0.005
	880 vs. 980 m	0.084	0.175	0.0453	0.263	1.1243	0.325

**TABLE 5:** Dissimilarity test of phyllosphere fungal community between two different sample groups among different elevations at the seedling, heading, and mature stages.

Growth stage		MRPP		ANOSIM		PERMANOVA	
		Delta	*p*	*R*	*p*	*F*	*p*
	580 vs. 680 m	−0.007	0.613	0.024	0.311	0.061	0.517
	580 vs. 780 m	0.003	0.357	0.234	0.110	0.106	0.194
	580 vs. 880 m	0.010	0.233	0.062	0.210	0.090	0.160
	580 vs. 980 m	0.039	0.032	0.220	0.031	0.131	0.044
Seeding	680 vs. 780 m	0.011	0.197	0.201	0.104	0.128	0.120
stage	680 vs. 880 m	0.016	0.156	0.105	0.130	0.108	0.144
	680 vs. 980 m	0.045	0.026	0.267	0.017	0.155	0.026
	780 vs. 880 m	0.022	0.146	0.259	0.081	0.147	0.061
	780 vs. 980 m	0.000	0.407	0.114	0.231	0.107	0.348
	880 vs. 980 m	0.004	0.349	0.049	0.229	0.089	0.293
	580 vs. 680 m	0.121	0.069	0.500	0.029	0.267	0.119
	580 vs. 780 m	0.630	0.031	1.000	0.028	0.861	0.030
	580 vs. 880 m	0.383	0.029	0.979	0.025	0.667	0.027
	580 vs. 980 m	0.524	0.024	1.000	0.031	0.827	0.035
Heading	680 vs. 780 m	0.412	0.032	0.583	0.039	0.558	0.032
stage	680 vs. 880 m	0.191	0.059	0.552	0.057	0.413	0.064
	680 vs. 980 m	0.276	0.029	0.583	0.026	0.474	0.023
	780 vs. 880 m	0.505	0.032	0.885	0.034	0.689	0.029
	780 vs. 980 m	0.677	0.030	0.458	0.019	0.904	0.027
	880 vs. 980 m	0.182	0.031	0.500	0.029	0.357	0.058
	580 vs. 680 m	0.269	0.023	0.375	0.024	0.360	0.020
	580 vs. 780 m	0.308	0.026	0.531	0.031	0.440	0.023
	580 vs. 880 m	0.124	0.157	0.125	0.278	0.232	0.182
	580 vs. 980 m	0.145	0.064	0.448	0.057	0.298	0.042
Mature	680 vs. 780 m	0.529	0.025	1.000	0.023	0.822	0.029
stage	680 vs. 880 m	0.253	0.028	0.542	0.019	0.296	0.029
	680 vs. 980 m	0.301	0.023	0.417	0.034	0.404	0.032
	780 vs. 880 m	0.163	0.037	0.427	0.020	0.226	0.052
	780 vs. 980 m	0.114	0.122	0.292	0.110	0.146	0.378
	880 vs. 980 m	−0.004	0.447	−0.021	0.481	0.103	0.575

At the seedling stage, the phyllosphere fungal communities at 980 m asl were significantly different from those at 580 and 680 m asl, respectively, and no significant differences were observed between the samples at other elevations (Table 5). At the heading stage, the phyllosphere fungal community at 680 m asl was not significantly different from those at 580 and 980 m asl, while significant differences were observed between other elevations. At the mature stage, the phyllosphere fungal communities at 580 m asl were significantly different from those at 680 and 780 m asl; the phyllosphere fungal communities at 680 m were significantly different from those at 780, 880, and 980 m asl; the phyllosphere fungal communities of rice samples at 780 m asl were significantly different from those at 880 m asl; and there were significant differences between the other elevations.

### Relationships Among Physicochemical Properties and Microbial Communities

**FIGURE 6:**
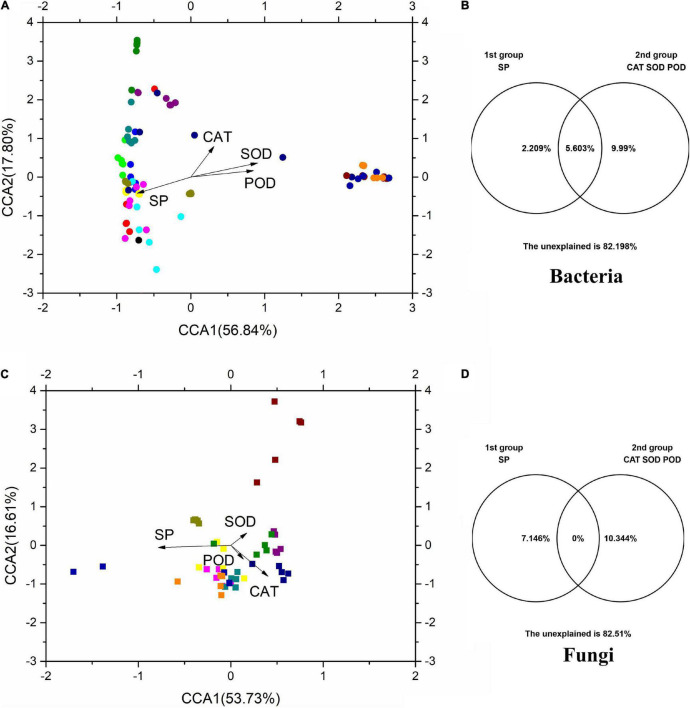
The CCA and VPA of rice phyllosphere bacteria **(A,B)** and fungi **(C,D)** among different elevational sites and growth stages. CCA, canonical correspondence analysis; VPA, variance partitioning analysis. SP, soluble protein; CAT, catalase; SOD, superoxide dismutase; POD, peroxidase.

Canonical correlation analysis was used to investigate the relationships between microbial communities and physicochemical properties (Figure 6). CCA1 and CCA2 accounted for 56.84 and 17.80%, respectively, of the total variation in the bacterial communities (Figure 6A), while accounting for 53.73 and 16.61%, respectively, of the total variation in the fungal communities (Figure 6B). The results of VPA showed that SP and antioxidant enzymes (CAT, SOD, and POD) accounted for 2.21 and 9.99%, respectively, of the total variation of phyllosphere bacterial populations (Figure 6). In addition, SP and physicochemical properties accounted for 7.15 and 10.34% of the total variation of phyllosphere fungal populations, respectively. These physicochemical properties also, respectively, accounted for 12.20 and 17.49% of the total variation in the bacterial and fungal communities. Among these physicochemical properties, SP shared a strong positive correlation with bacterial and fungal communities (*p* < 0.05) based on the Bray–Curtis distance and Jaccard distance matrix (Supplementary Table 1).

**FIGURE 7:**
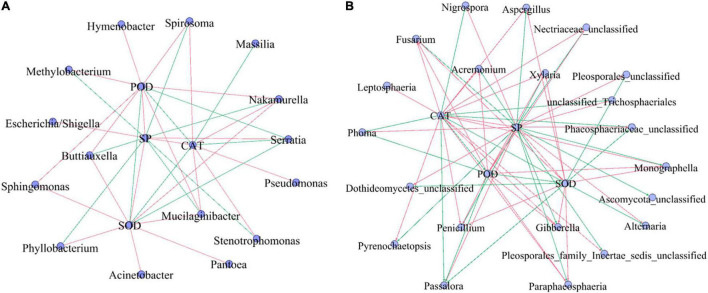
The correlation analysis among SP, antioxidant enzyme (SOD, POD, and CAT), and top 30 bacterial **(A)** and fungal **(B)** populations. SP, soluble protein; CAT, catalase; SOD, superoxide dismutase; POD, peroxidase. Red line: negative correlation, green line: a positive correlation. The correlation among SP, antioxidant enzyme (SOD, POD, and CAT), and top 30 microbial populations (genera) by the Pearson methods at *p* < 0.05. Standardization method: standardize environmental data only (scale each factor to zero mean and unit variance).

Correlation analysis of SP, antioxidant enzymes (SOD, POD, and CAT), and the top 30 dominant microbial genera (Figure 7) showed that SP showed a strong significantly negative correlation with *Serratia*, *Passalora*, unclassified_Trichosphaeriales, antioxidant enzyme (*R* > 0.5, *p* < 0.05), and significantly positive correlation with the fungal genera *Xylaria*, *Gibberella*, and *Penicillium* (*R* > 0.5, *p* < 0.05). Among the top 30 bacterial genera, *Mucilaginibacter* and *Nakamurella* had significant negative correlations with antioxidant enzymes, but weak correlations (*R* < 0.5, *p* < 0.05). *Serratia* had a strong significant positive correlation with antioxidant enzymes (*R* < 0.5, *p* < 0.05). Among the top 30 fungal genera, *Fusarium*, *Gibberella*, *Monographella*, *Paraphaeosphaeria*, *Penicillium*, and *Xylaria* had significant negative correlations with antioxidant enzymes (*R* < 0.5, *p* < 0.05), while *Passalora* had a strong significant positive correlation with antioxidant enzymes (*R* > 0.5, *p* < 0.05).

## Discussion

The leaves of plants present an area that is available for potential microbial colonization and growth, and the microbial populations of this phyllosphere can be abundant and taxonomically diverse (Bodenhausen et al., 2014). These organisms are crucial for leaf biological processes and ecosystem functions (Ortega et al., 2016), and have distinct impacts on their host plants by serving as plant pathogens or growth-promoting bacteria (Kinkel, 1997; Whipps et al., 2008). Microbial colonization of the phyllosphere is controlled by natural environmental factors. The variation of phyllosphere bacterial communities along elevational gradients may provide a potential link with temperature, which corresponds to elevation gradients over short geographic distances. At the same time, the plant growth stage is also an important factor in affecting phyllosphere microorganisms. Therefore, it is crucial to study the influence of both elevation gradients and growth stages on phyllosphere microorganisms, especially the core microbiome. In our study, we observed the dynamic change of phyllosphere microbial communities using an Illumina MiSeq-based approach, at different growth stages and elevations.

At all elevations, the rice leaf SP content decreased gradually with the rice growth stage. But at the same growth stage, there were slight differences in the SP between different elevations (Figure 1A). The results indicated that SP was less affected by elevation than it was more affected by the growth stage. The SP content was also an important factor affecting rice phyllosphere microorganisms (Figure 6). In addition, SP had a strong significant negative correlation with antioxidant enzymes (*R* > 0.5, *p* < 0.05).

The CAT, POD, and SOD are important antioxidant enzymes that cooperate to remove excess reactive oxygen species (ROS), thereby protecting the structures and functions of cellular components (Zhang et al., 2014). POD is widely distributed in plant tissues and is involved in diverse growth, development, and senescence processes in plants. CAT catalyzes the dismutation of excess H_2_O_2_ into oxygen and water, maintaining H_2_O_2_ at a low level, while SOD catalyzes the dismutation of superoxide into oxygen and hydrogen peroxide (Zhang et al., 2014). There was little difference in the activity of antioxidant enzymes at the seedling and heading stages; however, there was a great difference at the mature stage in the activity of these enzymes at different elevations (Figures 1B–D). This indicated that the activity of antioxidant enzymes in rice leaves was less affected by elevation at the seedling stage and heading stages, but was more affected by elevation at the mature stage. At the same elevation, the activity of antioxidant enzymes at the heading and mature stages was significantly higher than that at the seedling stage. These results indicated that the activity of antioxidant enzymes increased gradually during the growth process of rice and could be affected by elevation at the mature stage. In addition, *Serratia* and *Passalora* have strong significant positive correlations with antioxidant enzymes, which may be one of the causes of the changes in antioxidant enzymes.

There is a link between biodiversity and stability, as high biodiversity is generally associated with greater stability in an ecosystem. Some studies have suggested that the strength of species interaction determines the biodiversity and stability of the microbial community (Ratzke et al., 2020). In addition, other studies found that the biodiversity of keystone phylotypes determines crop production (Fan et al., 2021). In our study, from 680 to 980 m asl, there was no significant difference between the seedling and heading stages of rice, while diversity at the mature stage was significantly different from the two proceeding stages (Figure 2). Rice phyllosphere bacterial community α diversity showed different changes during the growth period at low (580 m), medium (680 and 780 m), and high (880 and 980 m) elevations (Figure 2). In addition, the variation trend of diversity at the seedling and heading stages were similar for different elevations. On the whole, diversity increased gradually with elevation, while diversity decreased gradually from 680 to 980 m asl at the mature stage (Figure 2). The fungal α diversity of rice phyllosphere first increased and then decreased with growth, with the highest α diversity observed at the heading stage, while the elevation had only a small effect on the α diversity of the rice phyllosphere fungal community (Figure 3). This indicated that the elevation had a greater effect on the α-diversity of phyllosphere bacteria than on fungi, and the growth stage had a great effect on both bacteria and fungi. The present results are in agreement with previous studies (Cordier et al., 2012a; Qian et al., 2018) that showed microbial diversity was affected by elevation and growth stages. Plant phyllosphere microorganisms associated with plant growth are very important to plant health and are closely related to important issues such as efficient utilization of nutrients, continuous cropping, and crop rotation. In general, the α diversity of rice phyllosphere bacteria displayed a more significant variation with elevation, which indicated that the influence of elevations on the α diversity of rice phyllosphere bacteria was higher than that of the growth stage. However, the variation of α diversity of rice phyllosphere fungi was not significant with elevation. On the contrary, at the same elevation, the variation of α diversity of rice phyllosphere fungi at different growth stages was more significant, which indicated that the growth stage had a greater effect on α diversity of rice phyllosphere fungi.

Phyllosphere microorganisms are strongly influenced by various biological and abiotic factors (Bulgari et al., 2011; Rastogi et al., 2012; Trivedi et al., 2012). However, phyllosphere-adapted bacteria may modify their local environment and increase the absorption of nutrients from the external environment (Bodenhausen et al., 2014). Previous research has indicated that phyllosphere fungal assemblages have a spatial structure that might be shaped by abiotic factors, especially temperature (Jumpponen and Jones, 2009; Cordier et al., 2012b). The variation of phyllosphere bacterial communities along elevation gradients may provide a potential link with temperature, which corresponds to elevation gradients (Cordier et al., 2012a). Our results showed that the composition of the phyllosphere bacterial and fungal communities varied considerably over a gradient at the same stage of rice growth, the most significant change occurred at 780 m asl (Figure 4). Previous studies have shown that the composition of phyllosphere fungal assemblages of European beech (*Fagus sylvatica*) also vary significantly along an elevation gradient, and the variations in the assemblage composition were well correlated with variations in the average temperature (Cordier et al., 2012a). In our study, the variation of phyllosphere bacterial and fungal communities during the same growth period may be due to the change in temperature caused by elevation. In addition, we also analyzed the composition structure of the phyllosphere microflora in the seedling, heading, and mature stages of rice growth at the same elevation by dissimilarity and PCoA. There was a significant difference in bacterial and fungal community composition at different growth stages. The bacterial community structure at the seedling and heading stages was similar but different from that at the maturity stage. However, there were significant differences in fungal community structure between the seedling stage, heading stage, and mature stage. The phyllosphere microbiome has been known to be affected by the host genotype, where the plant host genetic factors shape the associated microbiota (Bodenhausen et al., 2014), and the host genotype is an important determinant of crop health (Rumakanta et al., 2015).

Among the five elevations during the three growth stages, Proteobacteria, Actinobacteria, and Bacteroidetes were the dominant bacterial phyla, and Ascomycota and Basidiomycota were the dominant fungal phyla (Figure 4). However, the relative abundance and composition of the dominant bacterial phyla were affected differently at the various elevations, indicating that the main rice phyllosphere bacterial population was affected by elevation. Therefore, we further studied the relationship between the main phyllosphere bacterial genera and elevation, discovering that some bacterial genera displayed significant correlations. The results suggested that elevation was significantly and positively correlated with *Lapillicoccus* and *Methylobacterium* at the seedling and heading stages and negatively correlated with these two genera at the mature stage. Elevation was also significantly positively correlated with *Pantoea*, which can act as a pathogen-causing infection in plants and humans, at the seedling stage, but negatively correlated at the heading and mature stages. As the most common community members of the plant phyllosphere, *Methylobacterium* plays a critical role in protecting the host plants from various pathogens (Madhaiyan et al., 2006; Ardanov et al., 2012). *Alternaria* is a strong pathogenic fungus, which can cause a variety of plant diseases (Maiti et al., 2007; Logrieco et al., 2009; Kgatle et al., 2018). The relative abundance of *Alternaria* was positively correlated with elevation, which indicated that the higher the elevation, the greater the risk of disease in rice. At the heading stage, elevation was significantly negatively correlated with *Passalora*, Mycosphaerellaceae_unclassified, *Periconia*, etc., while at the mature stage the elevation was significantly positively correlated with *Passalora*, Mycosphaerellaceae_unclassified, *Periconia*, and so on, indicating that the elevation at the maturity stage and the heading stage had opposing effects on the phyllosphere fungal community.

## Conclusion

In summary, using high-throughput sequencing methods, this study demonstrated a significant shift in the diversity and community composition of phyllosphere bacteria and fungi at the rice seedling, heading, and mature stages along an elevational gradient from 580 to 980 m asl. The results showed that the elevation had a greater effect on the phyllosphere bacterial α diversity, but less on the α diversity of phyllosphere fungi. The growth stage had a great effect on the α diversity of both phyllosphere bacteria and fungi. The results also showed that the diversity and composition of phyllosphere bacterial and fungal communities varied significantly between elevations at different growth stages, in terms of both the relative abundance of species and the variations in bacterial and fungal composition were well correlated with variations in the average elevation. SP had a strong significant negative correlation with antioxidant enzymes (*R* > 0.5, *p* < 0.05). In conclusion, elevation and growth stage had significant effects on the diversity, composition, and abundance of rice phyllosphere bacterial and fungal communities.

## Data Availability Statement

The datasets presented in this study can be found in online repositories. The names of the repository/repositories and accession number(s) can be found below: https://www.ncbi.nlm.nih.gov/, PRJNA675674.

## Author Contributions

All authors listed have made a substantial, direct, and intellectual contribution to the work, and approved it for publication.

## Conflict of Interest

The authors declare that the research was conducted in the absence of any commercial or financial relationships that could be construed as a potential conflict of interest.

## Publisher’s Note

All claims expressed in this article are solely those of the authors and do not necessarily represent those of their affiliated organizations, or those of the publisher, the editors and the reviewers. Any product that may be evaluated in this article, or claim that may be made by its manufacturer, is not guaranteed or endorsed by the publisher.

## Footnotes

1
http://mem.rcees.ac.cn:8080

2
http://drive5.com/uparse/

## Supplementary Material

Supplementary Figure 1 The shared and united operational taxonomic unit (OUT) among different gradients and growth stages. B, bacteria; F, fungi. M, seedling stage; G, heading stage; S, mature stage. (A) 580 m asl; (B) 680 m asl; (C) 780 m asl; (D) 880 m asl; (E) 980 m asl.
