# Supplementary figures and images for: Scale-Dependent Effects of Growth Stage and Elevational Gradient on Rice Phyllosphere Bacterial and Fungal Microbial Patterns in the Terrace Field
Source: Front Plant Sci. 2022 Jan 14;12:766128. doi: 10.3389/fpls.2021.766128 (PMC8794795; doi:10.3389/fpls.2021.766128)

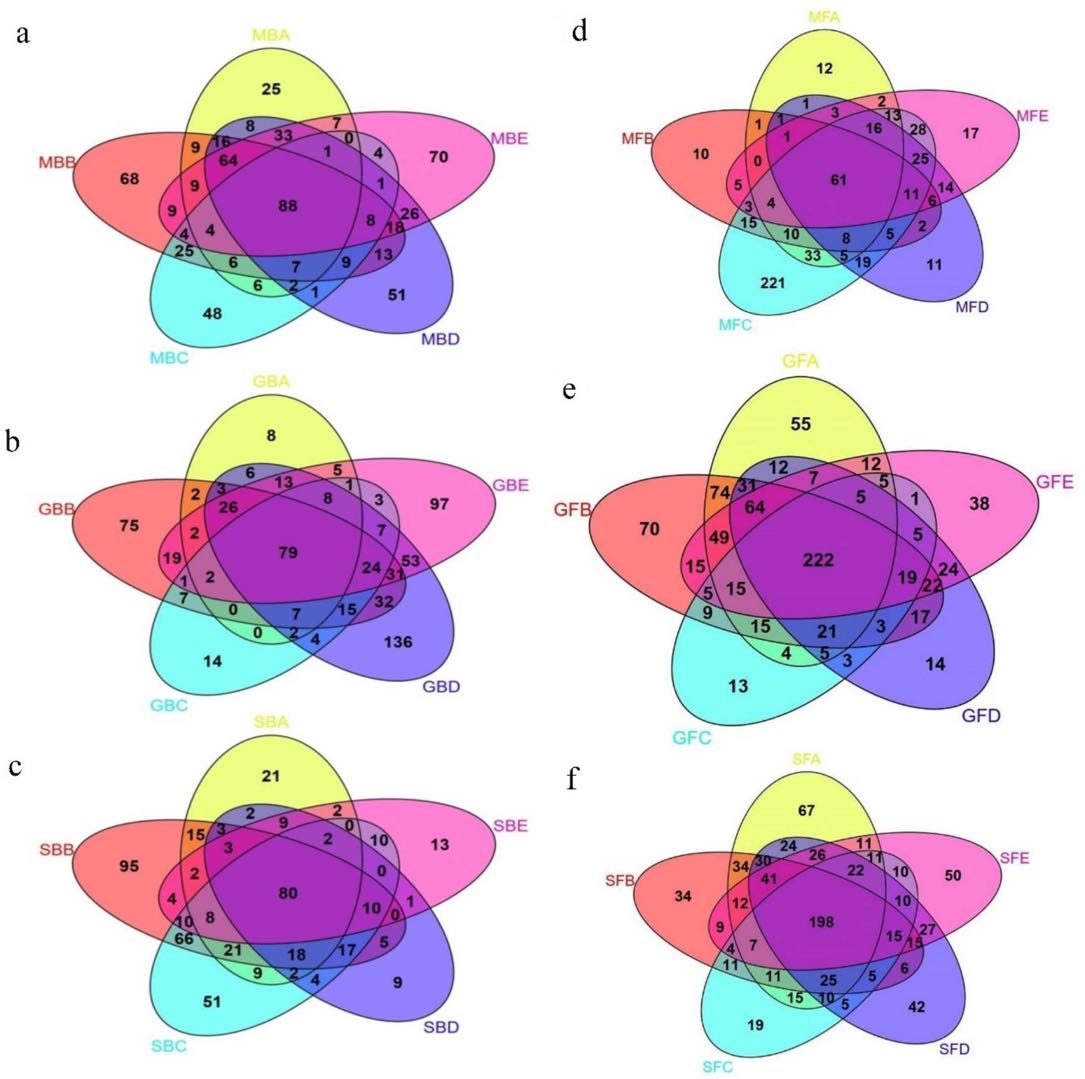

Supplement: Supplementary Figure 1 — The shared and united operational taxonomic unit (OUT) among different gradients and growth stages. B, bacteria; F, fungi. M, seedling stage; G, heading stage; S, mature stage. (A) 580 m asl; (B) 680 m asl; (C) 780 m asl; (D) 880 m asl; (E) 980 m asl. [file Image_1.JPEG]
