# Supplementary material for: Scale-Dependent Effects of Growth Stage and Elevational Gradient on Rice Phyllosphere Bacterial and Fungal Microbial Patterns in the Terrace Field
Source: Front Plant Sci. 2022 Jan 14;12:766128. doi: 10.3389/fpls.2021.766128 (PMC8794795; doi:10.3389/fpls.2021.766128)
Supplement: Supplementary file 2 [file Table_1.DOCX]

**Table S1 | Mantel analysis on the relationship between the relative abundance of OTUs and environmental factors.** BC: Bray-Curtis distance, JC: Jaccard distance. SP: soluble protein, CAT: Catalase, SOD: superoxide dismutase, POD: peroxidase.

| **Tax** | **Physicochemical properties** | **r(BC)** | **R(JC)** |
| --- | --- | --- | --- |
| **Bacteria** | **SP** | 0.2115*** | 0.2462*** |
|  | **CAT** | 0.0457 | 0.072 |
|  | **SOD** | 0.3072*** | 0.12 |
|  | **POD** | 0.2381*** | 0.168 |
| **Fungi** | **SP** | 0.6028*** | 0.6278*** |
|  | **CAT** | 0.4580*** | 0.4111*** |
|  | **SOD** | 0.0570 | -0.0092 |
|  | **POD** | 0.1074* | 0.0648 |
